# Bowel Resection and Ileotransverse Anastomosis as Preferred Therapy for 15 Typhoid Ileal Perforations and Severe Peritoneal Contamination in a Very Elderly Patient

**DOI:** 10.1155/2017/9424237

**Published:** 2017-12-21

**Authors:** Benjamin Momo Kadia, Desmond Aroke, Martin Hongieh Abanda, Tsi Njim, Christian Akem Dimala

**Affiliations:** ^1^Foumbot District Hospital, Foumbot, Cameroon; ^2^Grace Community Health and Development Association, Kumba, Cameroon; ^3^Health and Human Development (2HD) Research Network, Douala, Cameroon; ^4^Mbengwi District Hospital, Mbengwi, Cameroon; ^5^Non-Communicable Disease Unit, Clinical Research Education, Networking and Consultancy (CRENC), Douala, Cameroon; ^6^Bafang District Hospital, Bafang, Cameroon; ^7^Centre for Tropical Medicine and Global Health, Nuffield Department of Medicine, University of Oxford, Oxford, UK; ^8^Faculty of Epidemiology and Population Health, London School of Hygiene and Tropical Medicine, London, UK; ^9^Department of Orthopaedics, Southend University Hospital, Essex, UK

## Abstract

Typhoid ileal perforation (TIP) is the most lethal complication of typhoid fever. Although TIP is a surgical emergency by consensus, there is still much controversy regarding the most appropriate surgical approach to be used. Bowel exteriorization and secondary closure are usually recommended for patients presenting late with multiple TIPs and heavy peritoneal soiling. We, however, discuss a unique case of an 86-year-old patient with 15 typhoid ileal perforations successfully treated with one-step surgery comprising bowel resection and ileotransverse anastomosis in a resource-constrained setting of Cameroon.

## 1. Introduction

Typhoid fever is a severe infectious disease caused by the Gram-negative enteric bacillus *Salmonella typhi* [1, 2]. Intestinal perforations are the most frequent cause of typhoid-related morbidity and mortality [2–7]. These lesions commonly occur in the ileum [8]. Typhoid ileal perforation (TIP) is the most lethal complication of typhoid fever [3, 9, 10]. The high case fatality of TIP in low-income countries where typhoid is endemic makes the condition a public health menace in these settings [5, 11]. Mortality rates from TIP vary between 5 and 60% [9, 12]. In Cameroon, a recent cohort study conducted in two regional hospitals revealed that TIP was most frequently associated with peritonitis-related mortality [13].

Although TIP is a surgical emergency by consensus, the most appropriate surgical approach to be used remains controversial [5, 14, 15]. Generally, the appropriate surgical option for managing TIP is contingent on the general state of the patient, the site and number of perforations, and the extent of peritoneal contamination. Closure of the perforation with fresh edges or wedge resection of the ulcer area and closure are recommended for simple perforation with minimal peritoneal soiling. Bowel resection with or without anastomosis and closure of the perforation followed by ileotransverse anastomosis are best reserved for multiple perforations [8]. The recommended options for severely ill patients who present late with heavy peritoneal contamination are ileostomy [6, 15] and laparostomy [8] followed by secondary closure. In low-income countries, however, most severely ill patients are managed in resource-limited hospitals where suboptimal management prevails and TIP is invariably a fatality [5].

The aim of the current paper is to report an unusual case of an 86-year-old patient with 15 typhoid ileal perforations and significant peritoneal soiling successfully managed with one-step surgery comprising bowel resection and ileotransverse anastomosis in rural Cameroon. The clinical challenges presented by this rare and critical case in a resource-limited setting are also highlighted.

## 2. Case Presentation

An 86-year-old Cameroonian male was brought to our district hospital with complaints of fever for 4 weeks and abdominal pain and diarrhoea for 2 weeks. The fever was of low grade, intermittent, and without associated symptoms. He automedicated himself with suboptimal doses of amoxicillin, metronidazole, and ibuprofen tablets which were bought over the counter from a drug store. After 2 weeks of continuous use of these drugs, the fever was persistent and became associated with abdominal pain and diarrhoea. The pain was a mild burning intermittent hypogastric pain which was of gradual onset and with no relieving or aggravating factors. The diarrhoea was of gradual onset and started a few hours after the onset of abdominal pain. It was watery and intermittent, and he passed out small quantities of blood-stained yellowish stool about 4 times on average each day. He was taken to a traditional healer who gave him concoctions for 2 weeks during which there was progressive onset of a severe frontal throbbing headache, loss of appetite, and general weakness. The severity of the abdominal pain was increasing. He was then brought to our hospital after 4 weeks of illness.

His past history was significant for moderate arterial hypertension, which was controlled with hydrochlorothiazide. He frequently used nonsteroidal anti-inflammatory drugs for chronic low back pain. His past history was otherwise unremarkable.

On admission, the patient was wasted and prostrated and had sunken eyeballs. His Glasgow coma score was 15/15. His vital signs were: blood pressure 100/60 mm/Hg, pulse 124 beats/minute (regular, rapid, and thready), respiratory rate 29 breaths/min and regular, and temperature 39°C. The capillary refill time was 3 seconds. His conjunctivae were mildly pale, and his sclerae were anicteric. There was no palpable enlarged lymph node. His buccal cavity was dry. The abdomen was symmetrically distended and moved slightly with respiration. There was guarding and rebound tenderness at the hypogastrium. Digital rectal examination was without particularity but for an empty rectum. Bowel sounds were faint and hypoactive. The rest of the physical examination was normal.

In view of these, a diagnosis of localized peritonitis with severe sepsis was made. Aggressive fluid resuscitation with crystalloids was started. The patient was put on nil per os, and a nasogastric tube was inserted. Copious thick greenish fluid was brought out by the nasogastric tube. A urinary catheter was placed, and the urine output was monitored. The patient was placed on intravenous ceftriaxone (1 g, 8 hourly), gentamicin (160 mg daily), metronidazole (500 mg, 8 hourly), and paracetamol (1 g, 8 hourly). The following laboratory investigations were requested:

1. An erect chest X-ray which revealed air beneath the right hemidiaphragm and distended small-bowel loops.
2. The Widal test which was positive with a titre of <1/160 for both somatic and flagellar antibodies.
3. White cell count that showed leucocytosis at 16,000 cells/mm^3^ with 72% neutrophils.
4. Haemoglobin level which was 11.8 g/dL.

Blood cell indices were not fully assessed because our hospital could not do a complete blood count. Based on the historical, clinical, and laboratory data obtained, we considered intestinal perforation due to typhoid fever as the probable aetiology of peritonitis, and the aetiological differentials included perforated peptic ulcer and perforated appendix. However, we could not perform blood or stool culture in our primary care hospital to confirm typhoid fever.

**Figure 1:**
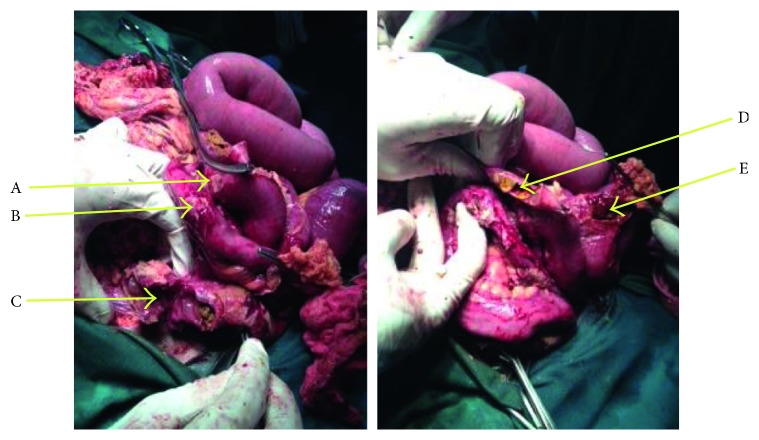
Intraoperative view of multiple typhoid ileal perforations (A to E) in the patient.

Four hours after admission and continuous resuscitation, the vital signs were blood pressure 120/80 mmHg, pulse 98 beats/min (regular and bounding), respiratory rate 24 breaths/min, and temperature 37.5°C. The patient remained haemodynamically stable the following 3 hours. Urine output was 32 ml/hr. His physical status score at the time of surgery was American Society of Anaesthesiology class IV. Intravenous ceftriaxone alongside other preoperative medication was administered to the patient, and an emergency exploratory laparotomy through a vertical midline incision was performed under general anaesthesia. Intraoperatively, distended small-bowel loops denuded of serosa on multiple spots were observed. Abundant greenish peritoneal fluid mixed with exudate and faecal material was obvious. The operative wound was rated as Altemeier class IV. On further inspection, multiple perforations were observed in the ileum (Figure 1).

**Figure 2:**
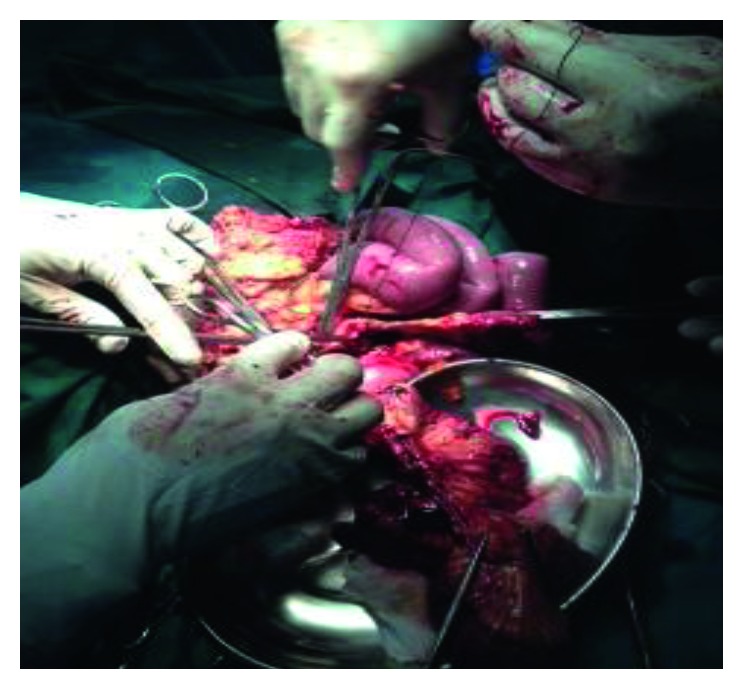
Segmental resection of the distal ileum.

A total of 15 perforations scattered over about 26 cm of the distal ileum were found. The perforation closest to the ileocaecal junction was less than 2 cm from the junction. The peritoneal cavity was thoroughly cleaned with warm saline. About 28 cm of the distal ileum including the section with perforations was segmentally resected (Figure 2) to leave clean margins, and then, a manual 2-layer end-to-side anastomosis of the proximal edge of the ileum and the transverse colon was performed.

**Figure 3:**
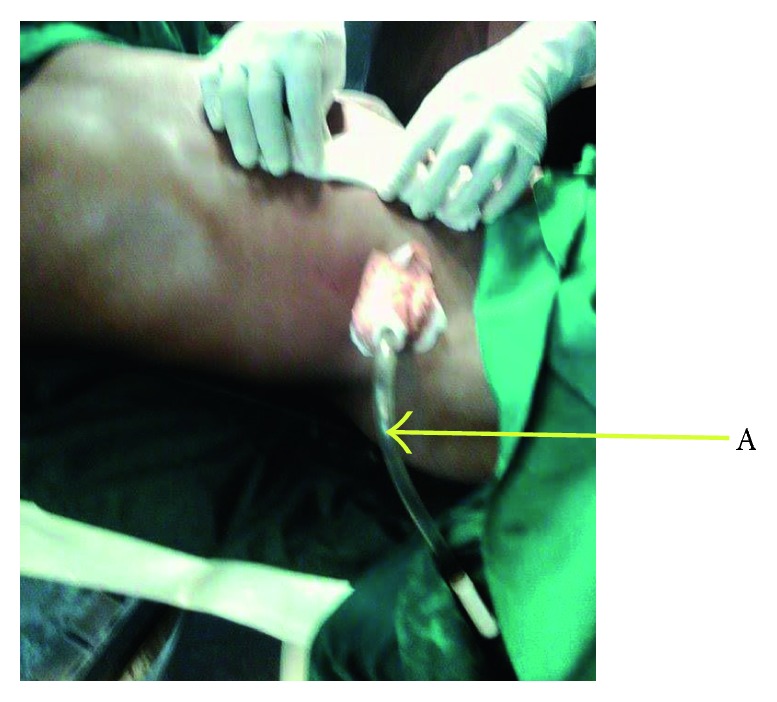
Abdominal drain (A).

The distal edge of the ileum was closed as a stump over the caecum, and the ascending colon left as a blind loop. Two abdominal drains were inserted to drain both the paracolic gutters and the rectovesical pouch through the right (Figure 3) and left lower quadrants of the abdomen.

The abdomen was closed layer by layer. Portions of the resected ileum were sent for histopathological analysis in a tertiary hospital. The patient was monitored in the recovery room for 30 minutes during which he remained haemodynamically stable. He was sent to the ward where close monitoring was continued.

Postoperatively, the patient was maintained on intravenous infusions of dextrose-saline, as well as intravenous ciprofloxacin (400 mg, 12 hourly), metronidazole (500 mg, 8 hourly), omeprazole (40 mg, 24 hourly), and intramuscular diclofenac (150 mg daily) for 10 days. Early ambulation was encouraged upon recovery from general anaesthesia. Enteral feeding with fluid diet was started after 26 postoperative hours, and routine postoperative care in the ward was continued. The abdominal drains were removed on the 6th postoperative day. On the 8th postoperative day, bowel sounds could not be perceived. A probable diagnosis of the postoperative paralytic ileus was made. The nasogastric tube was maintained, and enteral feeding halted till the tenth postoperative day when bowel sounds were appreciated. On the tenth postoperative day, suppuration was noticed at the surgical site which necessitated removal of the stitches and opening of the wound at the bedside. The wound was dressed for 3 weeks. Serial (four) Widal tests done at 3 days' interval remained positive with a titre of <1/160 for both somatic and flagellar antibodies. Histopathology of the resected intestinal tissue revealed diffuse aggregates of enlarged irregular pale cells, with eccentric nuclei, and abundant acidophilic cytoplasm, with phagocytic characteristics. Chronic inflammation of the Peyer patches was also noted. These findings confirmed that the multiple ileal perforations were due to typhoid fever. The patient was discharged home on the thirty-fifth postoperative day and scheduled for regular visits. No other complications were observed after 6 months of follow-up.

## 3. Discussion

TIP is associated with a characteristic acute abdominal pain which is rather of gradual onset in elderly patients [10]. Furthermore, the severities of the signs and symptoms of TIP do not depend on the multiplicity or sizes of the perforations [14]. These dissociations between TIP and expected clinical features could obscure the sinister course of the illness; delay diagnosis consequently leads to a high degree of peritoneal contamination and mortality in an elderly patient. A high index of suspicion in elderly patients is therefore imperative.

Delayed presentation of patients is a major hindrance to successful management of TIP in low-income countries. It results in significant changes in the ileum that require extensive surgery which further contributes to high morbidity and mortality [1, 8]. Our report is unique in that it is unusual for a frail and comorbid patient of such advanced age, and invariably depleted physiologic reserves, with multiple negative prognostic factors (including delayed presentation, sepsis, and up to 15 ileal perforations) to have a favourable surgical outcome.

Blood, stool, or bone marrow cultures are the most reliable standards of diagnosing typhoid fever. However, many low-income settings still rely on the Widal test to diagnose typhoid fever [16] which is considered an obsolete approach [8]. This was a major limitation in our preoperative diagnosis of typhoid fever. Diagnosis of intestinal perforation due to typhoid in resource-limited settings like ours is usually on the basis of (i) a history of a febrile illness possibly extending over several weeks, (ii) clinical data suggestive of generalized peritonitis, (iii) radiological data: X-ray showing air under the right hemidiaphragm, and (iv) intraoperative data: visualization of bowel perforations [1].

Generally, perforation occurs after severe inflammation and necrosis of the Peyer patches of the distal ileum [8, 17]. However, the characteristic inflammatory changes and lymphoid tissue involvement typical of typhoid fever require histological confirmation [9, 14], but this is beyond the reach of small hospitals like ours. Another possible aetiology of ileal perforation worth mentioning is frequent use of nonsteroidal anti-inflammatories. Multiple ileal perforations secondary to repeated parenteral diclofenac administration in an elderly patient have been reported [18] although the type of nonsteroidal anti-inflammatory drug and the route and frequency of drug administration differed from our case. Our initiative in sending out a specimen for histological analysis at a tertiary hospital permitted us to definitively confirm typhoid fever as the aetiology of ileal perforations in our patient.

From a treatment perspective, the need for enterostomy ought to be judiciously assessed in spite of clear indications as in our case. Our choice of operative procedure was justified by the known risk of morbidity of bowel exteriorization which is greatest in elderly patients, patients who present late, and patients undergoing emergency surgery. Furthermore, sepsis at presentation and the comorbid state of our patient further increased the risk of complications of bowel exteriorization [19]. Finally, the negative impact of poor stoma care in resource-limited settings cannot be overemphasized. Considering the likelihood of a damaged ileocaecal junction in our case, the option of ileocaecal anastomosis was dismissed since it would have been later on complicated by buildup of the colonic faecal material and toxins into the remaining ileum [20]. Also, primary closure was not a plausible option since most perforations in our case were large. However, considerable ileal resection as in our patient heralds potential complications like malabsorption, osmotic diarrhoea, and derangements in the bile salt metabolism [21]. The ascending colon had no perforations and was salvaged. Resecting this portion of the bowel in order not to leave a blind loop would have rendered the surgical procedure longer and increased the risk of morbidity and mortality in such a critical setting.

Previous case reports indicating more typhoid ileal perforations, albeit in relatively younger patients, indicate different surgical approaches: a case of 25 perforations involving the distal jejunum and entire ileum successfully treated with single-layer closure [22]; a patient with 24 ileal and caecal perforations treated with bowel resection and ileotransverse side-to-side anastomosis with proximal ileostomy [23]; and a child with 27 ileal and colonic perforations necessitating hemicolectomy [24]. From these reports and ours, it is noted that even though the surgical procedures were individualized, optimal perioperative management was common to all the cases. Ameh et al. compared 3 operative procedures in Nigeria (although amongst children) and proposed that although mortality was high following all the types of surgery, bowel resection and anastomosis was a preferred surgical treatment for typhoid perforation in low-income settings [25]. However, a recent study by Caronna et al. in Benin discourages bowel resection and anastomosis. It is, nonetheless, worth noting that Caronna et al. hugely attributed the high morbidity associated with resection and anastomosis to anastomotic complications [15], which based on our assessment did not occur in our patient and explains in part the good outcome. Whichever the surgical approach used, it is recommended to thoroughly search for other sites of perforation or necrosis that might imminently perforate [8]. In our case, apart from the perforations observed, denudation of the serosa was also noticed and was expected to heal spontaneously.

Postoperatively, the patient developed paralytic ileus and surgical site infection. Studies reviewing operative management of TIP in other resource-poor settings report surgical site infection as the most frequent postoperative complication of TIP repair [5, 26, 27]. In our case, postoperative complications were successfully treated conservatively. According to the Clavien and Dindo classification (a validated method of reporting adverse surgical events), these complications were of Grade I or least severe complications [28], but they prolonged the hospital stay of our patient.

In conclusion, the diagnosis and management of TIP in low-income countries that are endemic for typhoid fever are challenging. The successful treatment of an ominous case of multiple typhoid ileal perforations in a very elderly patient using a less conventional approach does not discount the high case fatality in resource-limited settings nor the merits of evidence-based therapeutic modalities. Highlighting the importance of early diagnosis and the utilization of existing health facilities may help dispel patient inertia and late presentation. These, in addition to individualized and optimal surgical care, may lead to a favourable outcome even amidst resource constraints.
